# PD153 Horizon Scanning Analysis Of The Obesity Medicines Pipeline

**DOI:** 10.1017/S0266462324003854

**Published:** 2025-01-07

**Authors:** Sola Akinbolade, Jane Nesworthy, Ross Fairbairn, Nicole O’Connor, Amy Hussain, Bethan Harris, Dawn Craig

## Abstract

**Introduction:**

Horizon scanning provides timely intelligence about innovative health technologies in clinical development by commercial and non-commercial organizations. The horizon scanning for obesity medicines, carried out by the National Institute for Health and Care Research Innovation Observatory (IO), aimed to identify emerging obesity medicines to inform decision-making by national stakeholders and to shape future research.

**Methods:**

In July 2023, the IO utilized horizon scanning methodology to identify medicines for preventing and treating obesity either primarily or as a comorbidity. The scans included medicines in preclinical and clinical development (phase I, I/II, II, II/III, III, or IV) sponsored by industry and non-industry for all population groups. Trial locations included Australia, Canada, the European Union, the UK, and the USA. Data were collected from the IO’s internal database (the Medicines Innovation Database), ClinicalTrials.gov, the European Union Drug Regulating Authorities Clinical Trials Database, the World Health Organization International Clinical Trials Registry Platform, and the Citeline Pharmaprojects database. The data were systematically screened and analyzed.

**Results:**

A total of 405 clinical trials were identified that evaluated 177 unique medicinal interventions. Among these, 47 unique preclinical interventions were identified from preclinical studies. A total of 256 (63%) trials were sponsored by industry, 139 (34%) by non-industry, and 10 (3%) by industry and non-industry jointly. The top five drug classes included anorectic or anti-obesity medicines (n=75; 42%), antihyperglycemics (n=24; 14%), anti-inflammatories (n=8; 5%), hepatoprotectants (n=7; 4%), and antihyperlipidemics (n=4; 2%). At the time of scanning, 48 (27%) medicines were unlicensed in the UK and 129 (73%) were not. Among the licensed medicines, 37 (77%) were off patent and 11 (23%) were on patent.

**Conclusions:**

The IO’s horizon scanning process can identify and deliver timely intelligence to support decision-making and facilitate adoption of new medicines to target areas of unmet clinical need. The obesity medicines scan identified medicinal interventions in preclinical and clinical development and provides valuable insights into the trends and research gaps in preventing and treating obesity.

